# Biomakers in Chronic Chagas Cardiomyopathy

**DOI:** 10.3390/microorganisms10081602

**Published:** 2022-08-09

**Authors:** Angela Braga Rodrigues, Henrique Oswaldo da Gama Torres, Maria do Carmo Pereira Nunes, Juliana de Assis Silva Gomes, Aline Braga Rodrigues, Laura Lopes Nogueira Pinho, Manoel Otavio Rocha, Fernando Antonio Botoni

**Affiliations:** 1 Postgraduate Course of Infectious Diseases and Tropical Medicine, The Post-Graduate Program in Infectious Diseases and Tropical Medicine (PPG-IMT), School of Medicine, Universidade Federal de Minas Gerais, Belo Horizonte 30130-100, MG, Brazil; 2 School of Medicine, Universidade Federal de Minas Gerais, Minas Gerais, Belo Horizonte 30130-100, MG, Brazil; 3 Department of Morphology, Institute of Biological Sciences, Universidade Federal de Minas Gerais, Belo Horizonte 31270-901, MG, Brazil; 4 Department of Cardiology, Hospital Felicio Rocho, Belo Horizonte 30110-934, MG, Brazil

**Keywords:** Chagas cardiomyopathy, cytokines, biomakers

## Abstract

The primary objective was to observe the relationship between serum levels of BNP, Ca-125, C-reactive protein and uric acid as prognostic and functional markers in patients with chronic Chagas cardiomyopathy (CCC). Circulating levels of cytokines: IL-1β, TNFα, IL-10, IL6, IL-8 and IL-12 were determined and investigated regarding their association with hemodynamic parameters, clinical signs of heart failure and outcome. Chagas is still a neglected disease that affects numerous individuals, many of them in their most productive years. CCC with left ventricular dysfunction is the most severe presentation of Chagas Disease. BNP is a well-recognized prognostic and clinical biomarker, not only in chronic heart failure patients but also in patients with CCC. Previous studies have shown Ca-125, C-reactive protein, and uric acid to be potentially good prognostic markers in heart failure (HF). Fifty patients with left ventricular fraction less (LVEF) than 55% were selected and followed for a mean period of 18 ± 8.3 months. Patient’s mean age was 43.42 ± 10.3 years (32 male), their BNP was 293 (160–530) pg/mL, Ca-125 8.5 (5.5–16.75) U/mL, uric acid 6.2 ± 2 mg/dL, and C- reactive protein 4.5 (4.5–7.3) mg/L. Patients who had LVEF less than 35% had higher BNP (*p* = 0.0023), Ca-125 (*p* = 0.027) and uric acid (*p* = 0.01) serum levels. Patients who died also showed higher BNP (*p* = 0.01), uric acid (*p* = 0.05) and a trend towards higher Ca-125 serum levels (*p* = 0.056). All markers: BNP, Ca-125, uric acid and C-reactive had good predictability of death in Cox-regression univariate analysis, however, not on the final multivariate model. Of the inflammatory cytokines, IL-8 and IL-12 showed a relation to LVEF of less than 35%. IL-12 was related to adverse cardiovascular events and non-survival. IL-1β was a good predictor of mortality in the final Cox regression model. Determination of Ca-125, uric acid levels and C-reactive protein may add useful clinical and prognostic information and may help clinical decision making for patients with CCC.

## 1. Introduction

Chagas disease is one of WHO neglected diseases affecting 6–7 million people, with most of them living in Latin America [1]. Recently, it has also reached the US, Europe and even Japan due to the migration of infected individuals from endemic areas. Although it is estimated that around 300,000 infected people live in the US, it remains poorly recognized and is considered one of the five neglected parasitic infections in North America, by the Center for Disease Control (CDC) [2].

Chronic Chagas cardiomyopathy (CCC) is a severe and progressive form of myocarditis and is the most important manifestation of Chagas disease. The disease affects individuals in their most productive years and has a high impact on morbidity and mortality, with an annual mortality rate of 0.2 to 19.2%, depending on the population studied [3,4].

The diagnosis of CCC comprises the detection of serum antibodies against *T cruzi* or its components by two different techniques (usually ELISA, indirect fluorescent assay, indirect haemagglutination), in addition to evidence of cardiac involvement assessed by the resting ECG or echocardiography [5].

CCC evolves into three main syndromes: heart failure, cardiac arrhythmia and thromboembolism [3]. It is a heterogeneous condition, ranging from asymptomatic individuals with mild ECG abnormalities to the more severe and morbid condition: dilated cardiomyopathy with left ventricular systolic dysfunction [3]. Left ventricular systolic dysfunction is the most important predictor of death in CCC [4].

CCC has notable clinical pleomorphism, some individuals will have mild electrocardiography and echocardiography abnormalities without significant symptoms. In other individuals, the disease progression will lead to refractory heart failure (HF). Some individuals will be asymptomatic but will die suddenly and not even reach middle age. The clinical course is so difficult to predict due to wide inter-individual variability. The determination of reliable and potent biomarkers could help individualize prognosis and define more aggressive management to achieve a better outcome [4,5,6].

Brain natriuretic peptide (BNP) is an important biomarker for heart failure (HF) diagnosis and prognosis [7]. In Chagas disease BNP serum levels were related to left ventricular dysfunction, to ventricular arrhythmias and were a good predictor of death [7,8,9,10]. Ca-125, uric acid and C-reactive protein were previously related to disease severity and outcome in patients with HF [11,12,13].

Therefore, we investigated if these parameters could work as functional markers and could also be predictors of adverse cardiovascular outcomes, in patients with CCC. We have compared these parameters with BNP and analyzed if they could be as potent. We aimed to verify if these simple and lower-cost markers that can be determined in most places could aid the clinician in the routine clinical care of patients with such a severe disease.

Serum levels of pro-inflammatory cytokines were also determined and evaluated in their relation to hemodynamic parameters, clinical signs of HF, and outcome.

## 2. Methods

50 patients were consecutively selected at our Reference Outpatient Center for Chagas Disease of the Federal University of Minas Gerais. Inclusion criteria consisted of two positive serology tests for Chagas disease and the presence of left ventricular ejection fraction (LVEF) less than 55%, and left ventricular end-diastolic diameter (LVED) greater than 56 mm on echocardiogram. Diseases such as diabetes, thyroidopathies, hypertension, and other etiologies of heart failure such as ischemic, valvular or idiopathic were excluded, as well as patients with the intestinal and cardiodigestive form of Chagas disease.

Patients were followed up for a mean period of 18 ± 8 months. The patients’ mean age was 53.4 (±10.3). During the first medical visit, participants underwent a complete clinical examination and NYHA was determined: NYHA I: 22/50 patients, NYHA II 15/50 patients, NYHA III 11/50 patients and NYHA IV 2/50 patients. Clinical examination included determination of arterial pressure, and heart rate, checking for pulmonary abnormal sounds and abdominal examination searching for ascites and hepatomegaly. Few patients had signs or symptoms of fluid congestion. Only 5 (10%) patients had hepatomegaly and ankle edema and only 6 (12%) had wheezing and 11 (22%) had lung crackles. Only one patient had pericardial effusion. Patients’ features are reported in Table 1.

Participants underwent a complete routine laboratory workup in our university laboratory, where Ca-125, uric acid and C-reactive protein serum levels were also measured.

Sampling of BNP and inflammatory cytokines was determined with tubes containing EDA which were processed for plasma extraction and then frozen at minus 40 degrees. BNP was processed using a high sensitivity extraction free radioimmunoassay kit (Triage Meter Plus kits—Penisula/Bachen laboratories, San Carlos, CA, USA). Cytokines were quantified by ELISA with a previously described method by Chen et al.

Patients were followed regularly at our clinic and their treatment was adjusted according to the current guidelines. Medications consisted of angiotensin-converting enzyme inhibitors or angiotensin receptor agonists (82%), beta-blockers (81.6%), diuretics (63.6%), glycosides (24%) and amiodarone (44%).

Echocardiograms were performed by the same professor on Cardiology using a Philips HDi 5000-ATL (Bothell, WA, USA) echo machine, using criteria established by the American Society of Echocardiography, and ejection fraction was accessed by the Simpson technique. Measured variables included: left ventricular end-diastolic diameter (LVEDD) and left ventricular end-systolic diameter (LVESD), using two-dimensionally guided M-mode. The left ventricular ejection fraction (LVEF) was calculated by the Simpson technique from the apical four-chamber view. On the pulsed Doppler of transmitral flow, peak velocity in early filling (E) and during atrial systole (A) and the deceleration time of early filling (DT) were measured. Right ventricular dilation (RV dilation) has been shown to be an important prognostic factor not only in patients with CCC but also in other etiologies of HF.

Patients were grouped according to hemodynamic parameters measured on echocardiogram (LVEF and RV dilation) to compare the markers’ serum levels between them and verify the presence of significant differences. Further, patients who had an adverse cardiovascular event were compared to the group of patients that remained event free, and the differences in the markers of clinical and hemodynamic variables were analyzed. The same investigation was performed for the groups of patients who survived and those that died.

The present study complies with the Declaration of Helsinki, and was approved by our local Ethics Committee. All patients signed a written informed consent.

## 3. Statistical Analyses

Statistical analyses were performed using the Statistical Package for Social Sciences (SPSS). Data are presented as mean plus standard deviation if variables were normally distributed and as median plus 25th and 75th percentiles if non-normal distributed. Differences between populations were accessed using the Student’s t-test or Mann-Whitney as appropriate. For categorical variables, results are reported as the percentage of total patients and Chi-square test was used to evaluate the relationship between them.

Cox proportional hazard analysis was used to assess the association between variables and mortality. Variables with *p* < 0.20 were found to be significant in the univariate analysis and were evaluated using stepwise multiple regression. For the multivariate model, variables were divided into three different blocks accordingly to clinical, echocardiography or laboratory categories. The final model was set with the most significant variables from each block and the final predictor was selected from them.

## 4. Results

Patients’ mean ejection fraction was 37.4 (±9.2), and 14 (28%) had RV dilation on echocardiogram. Patients’ features are reported in Table 1.

### Hemodynamic Parameters Measured by the Echocardiogram

**Figure 1:**
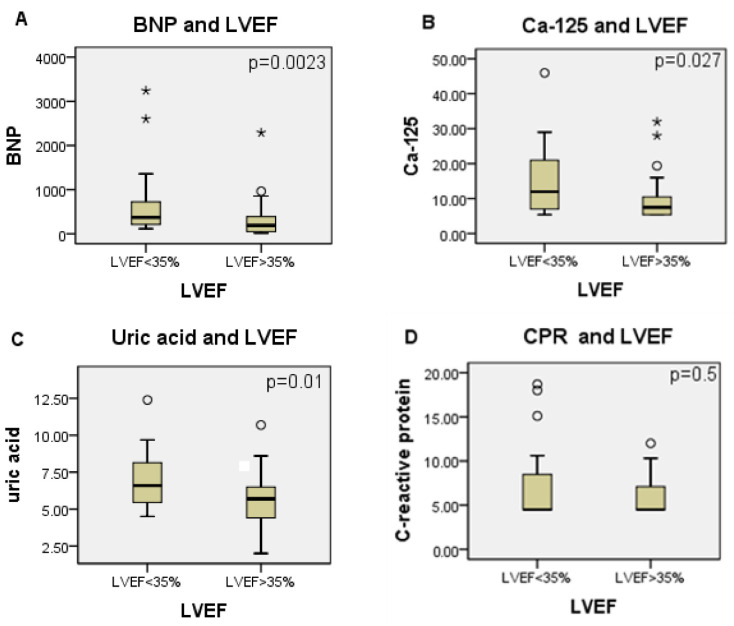
(**A**) BNP, Ca-125, uric acid, C-reactive protein serum levels and LVEF. (**B**) LVEF (patients grouped according to LVEF <35% or >35%) and BNP (bars represent median and 25th and 75th percentiles of circulating levels). (**C**) LVEF (patients grouped according to LVEF <35% or >35%) and Ca-125 (bars represent median and 25th and 75th percentiles of circulating levels). LVEF (patients grouped according to LVEF <35% or >35%) and uric acid (bars represent median and 25th and 75th percentiles of circulating levels). (**D**) LVEF (patients grouped according to LVEF <35% or >35%) and C-reactive protein (bars represent median and 25th and 75th percentiles of circulating levels). *, refers to group outliners; °, refers to group ouliners.

We observed that patients with LVEF of less than 35% had not only higher serum levels of BNP but also of uric acid and Ca-125. This data is presented in Figure 1.

**Figure 2:**
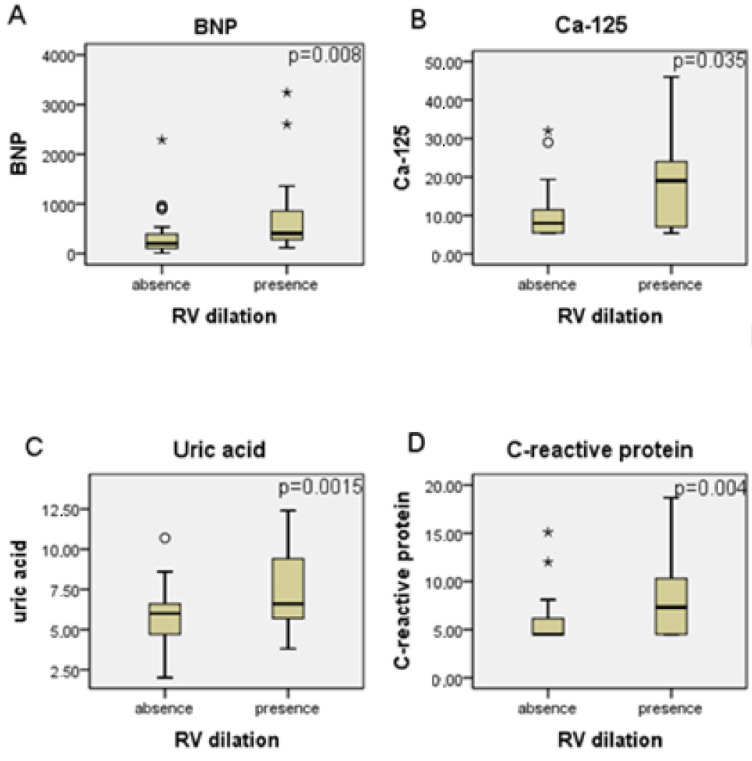
BNP, Ca-125, uric acid, C-reactive protein serum levels and RV dilation. (**A**) RV dilation (presence on echocardiogram) and BNP (bars represent median and 25th and 75th percentiles of circulating levels). (**B**) RV dilation (presence on echocardiogram) and Ca-125 (bars represent median and 25th and 75th percentiles of circulating levels). (**C**) RV dilation (presence on echocardiogram) and uric acid (bars represent median and 25th and 75th percentiles of circulating levels). (**D**) RV dilation (presence on echocardiogram) and C-reactive protein (bars represent median and 25th and 75th percentiles of circulating levels). *, group outliers; ° group outliers.

Patients grouped according to the presence of RV dilation on echocardiogram showed higher levels of BNP, ca-125, uric acid and C-reactive protein. This data is reported in Figure 2.

When analyzing the NYHA functional class, only BNP serum levels were higher in patients in the more advanced functional class (*p* = 0.005). Further, patients with higher Framingham scores had higher BNP levels (*p* = 0.003) and higher uric acid levels (*p* = 0.006).

We have measured pro-inflammatory cytokines: IL-1β, IL-6, IL-8, IL-10, IL-12, and TNFα Median serum values for our patient population are displayed in Table 1. We have verified if we could find any association between the cytokines and subgroup of patients with LVEF less than 35%, with the presence of right ventricular dilation on echocardiogram and with patients in more advanced NYHA functional class. Patients with LVEF less than 35% had higher IL-8 (*p* = 0.022) and IL-12 (*p* = 0.021) serum levels. These values are shown in Table 1. We could also observe an association between higher IL-8 serum levels and the presence of right ventricular dilation (*p* = 0.012) and more advanced functional class (*p* = 0.013). IL-6 was only related to the presence of right ventricular dilation on the echocardiogram (*p* = 0.01). IL-12 levels were higher in patients with more advanced functional class (*p* = 0.04) and patients with LVEF of less than 35% (*p* = 0.021). We did not observe any significant difference in IL-1β, TNFα, and IL-10 in the variables that were studied.

We have also determined procalcitonin circulating levels in CCC patients. Only 2 patients showed abnormally elevated values. One of them had severe disease and died, he had the highest level of 0.23. The other patient had a circulating level of 0.15. She had no adverse events. Both of them were in NYHA function class III and had RV dilation on the echo scan. None of them had suspected infection. The majority of patients had procalcitonin levels of 0.04.

Procalcitonin levels showed correlation with NYHA (*p* = 0.016) and RV dilation (*p*= 0.022). It showed a trend toward significance regarding Ca-125 levels (*p* = 0.054).

## 5. Outcome

During the observation period, we observed 18 adverse cardiovascular events, six patients died, five patients required in-hospital treatment due to arrhythmic events, one patient had a transient ischemic attack and six had progressively worsening HF.

Among the markers tested, only BNP levels were increased in patients that had an adverse cardiovascular event. Regarding cytokines, IL-8 and IL-12 showed higher levels in patients who suffered an adverse event. Among the hemodynamic parameters investigated, LVEDD, LVESD, LA index volume and right ventricular impairment were higher in patients with adverse cardiovascular events. In contrast, pulmonary systolic pressure (PSAP) and the ratio of early transmitral velocity to tissue Doppler mitral annular early diastolic velocity (E/e’) did not reach any significance. Clinical, echocardiography and laboratory parameters related to adverse events are reported in Table 2.

In the period studied we found a mortality of 12%, four patients died from sudden death and two from refractory HF. Patients who died had higher serum BNP levels (*p* = 0.01) and uric acid levels (*p* = 0.05) than those who survived. There was a trend towards higher levels of Ca-125 levels (*p* = 0.056) in non-survivals. Inflammatory cytokines had no significant differences between survivals and non survivals. The hemodynamic variables tested: LA index volume and right ventricular dilation on echocardiogram had significant difference between the groups. In contrast, LVEDD, LVEED, PSAP, and E/e’ did not show any significance. This is reported in Table 3.

To evaluate if the markers could predict death, a univariate and multivariate analysis by the Cox regression method was used, with three categories of variables divided according to clinical, laboratory and echocardiographic parameters.

In the univariate analyses, variables that reached a *p* < 0.20, were selected to enter the multivariate analyses. For the clinical category, the following variables were found to be significant (*p* < 0.20): functional class, abnormal pulmonary sounds, physical signs of right ventricular failure (hepatomegaly, ankle edema), radiological signs of venous congestion, and pulmonary rales. For the echocardiography category, the following variables were found to be significant (*p* < 0.20): right ventricular dilation, LVEDD, PSAP, and left atrial volume. In the laboratorial category: C-reactive protein, IL-1β, TNFα, Ca-125 were found to be significant (*p* < 0.20). This is reported in Table 4. BNP levels did not have statistical significance in the univariate model but it was included in the multivariate model owing to it relevant clinical meaning.

In the final multivariate model, only IL-1β was a significant predictor of death. The presence of pulmonary rales on physical examination showed a trend of significance. An interesting fact was that right ventricular dilation, a powerful event in the univariate analyses (its presence meant a six-time higher risk of death) lost its statistical significance in the presence of Ca-125 and Il-1 (a pro-inflammatory cytokine). This is reported in Table 4 and Table 5.

## 6. Discussion

It was observed that patients with LVEF of less than 35% had higher circulating levels of BNP, uric acid, and Ca-125. These markers were also increased in participants grouped according to the presence of right ventricular dilation on the echocardiogram. When analyzing the outcome, patients who died had higher serum levels of BNP and uric acid and showed a trend toward higher levels of Ca-125. However, only BNP serum levels were increased in patients who had adverse cardiovascular events.

CCC patients showed median BNP circulating levels of 254 (154–445) pg/mL and those patients who died had higher serum levels of BNP, median value of 790 (452–1063) pg/mL. In our study, BNP was the most powerful marker, not only concerning the hemodynamic parameters measured by the echocardiogram but also concerning the outcome and the severity of the clinical manifestations of CCC.

This is in line with previous studies which showed BNP to be a prognostic marker, in addition to its relation with left ventricular dysfunction and even to be associated with ventricular arrhythmias and diastolic dysfunction in CCC patients [7,8,9,10]. 

Ca-125, a glycoprotein that belongs to a family of mucins is a well-recognized marker for ovarian cancer, but many recent studies have reported its relation to HF [11,12]. Furthermore, some publications showed Ca-125 to have an association with NYHA class, congestive signs of HF, and even to be a predictor of death in HF non-chagasic patients [11,14,15,16,17,18]. The present investigation showed that in CCC patients, circulating Ca-125 levels were higher in those with LVEF of less than 35%. Moreover, Ca-125 was also increased in patients with right ventricular dilation, which is a known impaired prognostic predictor. Moreover, Ca-125 showed a trend towards higher levels in non-survivors and reached good predictability of mortality in Cox-regression univariate analysis but not on the final multivariate model.

The Ca-125 reference value for the diagnosis of ovarian malignancy is considered 35 U/mL. In the present study, patients’ median serum value was 9 (5.5–17.2)U/mL and for those who died was 20 (10.3–74) U/mL. Even though these values are lower than the threshold traditionally reported, the range of value with potential clinical implication in patients with HF remains unknown [16].

Uric acid, an end-product of purin metabolism, is a well-recognized serum marker for gout. Hyperuricemia association with cardiovascular disease has been described two centuries ago and clinical trials have demonstrated uric acid to be a marker of impaired vascular function and inflammatory cytokine activation [12,19,20]. A recent meta-analysis reported that elevated serum uric acid is associated with all cause and cardiovascular mortality [21]. In the present investigation, patients with more severe disease had higher circulating levels of uric acid, additionally, those who died showed elevated uric acid levels and it reached mortality prediction in the univariate analysis.

Critical levels of hyperurecemia in patients with HF varied in different previous studies, from 7.4 to 9.5 mg/dL [19,20]. In the present investigation, participants’ mean circulating levels of uric acid were 6.2 (±2) mg/dl and in those patients’ who died, the mean uric acid serum level was 7.7 (±2.8) mg/dL.

C-reactive protein, a nonspecific acute-phase protein produced by hepatocytes in response to IL-6 during inflammation and infection, has also been linked to HF in numerous studies and has been associated with a worse prognosis [13,22].

In our study, patients who died or who had an adverse cardiac event did not show elevated levels of C-reactive protein, however, this marker, reached good predictive ability in the univariate analysis.

In the present investigation, participants’ median C-reactive protein levels were 4.5 (4.5–7.3) mg/dL and in those patients that did not survive, C-reactive protein levels were 5.7 (4.5–18) mg/dL. Previous studies in patients with HF, showed C-reactive protein levels of 3.23 to 12 mg/dL to be related to risk of an adverse event or death [12,22].

Our results showed that of the inflammatory markers analyzed (TNFα, IL-1β, IL-6, IL-8, IL10, and IL-12) only IL-8 and IL-12 had higher levels in patients with LVEF of less than 35% Furthermore, patients with the presence of right ventricular dilation on echocardiogram showed elevated IL-6 and IL-8 levels.

IL-12 is a pro-inflammatory cytokine involved in T-helper (Th1) differentiation; it is linked to cardiac remodeling and coronary atherosclerosis. In our study, patients with worse outcome, more advanced functional class, and LVEF of less than 35% showed higher circulating levels of IL-12 [23,24].

Another work reported that patients with CCC had higher levels of IFN-γ, TNFα, and IL-6 when compared to individuals with the indeterminate form of the disease. However, patients with the indeterminate form had higher levels of IL-10 compared to CCC patients [25].

Although subgroups of patients with more severe heart disease and those with worse outcome did not show higher serum level, neither of IL-1 β nor of C-reactive protein, both these markers were important predictors in the Cox regression model. In the final analysis, IL-1β was the significant predictor. This is in line with an investigation that studied patients with idiopathic dilated cardiomyopathy, which also reported IL-1β to be the final predictor of death [26].

IL-1β is an apical proinflammatory cytokine that stimulates C-reactive protein production by induction of IL-6. HF is now recognized as a partially inflammatory disease with several hypotheses proposed to explain the origin of inflammatory cytokines. One of them proposes that a dysfunctional intestinal barrier would allow translocation of bacteria or its product into the circulation and stimulate cytokine production [27].

Pro-inflammatory cytokine IL-17 has also been implicated in CCC. A prior investigation suggested a protective role of IL-17 since it showed a direct correlation with cardiac function [28]. Further studies should examine the Th17 response by determining IL-17 and IL-23 circulating levels and analyzing them associatively with BNP.

Once ventricular dysfunction has developed, it carries an ominous prognosis in CCC patients, with mortality ranging from 0.2 to 19.2% depending on the population studied [4]. Chagas is still a neglected disease and affects numerous individuals in remote areas of Brazil. A recent cohort study has shown that these patients are still largely undertreated for HF [29]. The determination of simple markers such as Ca-125 and uric acid which have lower cost and wider availability than BNP and echocardiogram could help in clinical decision making and in the improvement of the medical management of CCC patients.

The determination and development of markers of cardiac malfunction can aid the clinician in the evaluation of prognosis, optimize management and individualize a more aggressive approach. This is especially needed for this neglected disease with such a variable clinical course that can lead to sudden death in asymptomatic individuals in their most productive years. 

## 7. Conclusions

Our results confirm that BNP is indeed a powerful clinical and prognostic marker in CCC patients. Ca-125 and uric acid showed increased serum levels in patients with left ventricular dysfunction and right ventricular dilation on echocardiogram. These markers also showed a link to survival. Functional and prognostic indicators are intensively investigated in HF. Markers of cardiac malfunction could be especially helpful in optimizing outcome in CCC, since it is a disease with striking individuality among affected patients. Additionally, it is a neglected disease, thus biomarkers with lower cost and that can be determined everywhere can offer an enormous help in the medical management of these cardiac patients that can, sometimes, have such a dismal prognosis.

IL-1β was shown to be a significant predictor of the outcome. The association of IL-1β circulating levels with mortality is important since the Il-1β axis is being investigated as a promising therapeutic target in HF patients [30] and maybe this can eventually be extended to CCC patients.

## Figures and Tables

**Table 1:** Patients Characteristics.

	Patients (n = 50)
Age (years)	53.4 (±10.3)
Sex (n male, %)	32 (64%)
Systolic blood pressure	102.6 (±13.3)
Heart rate (bpm)	61.6 (±7.2)
NYHA III/IV (n, %)	13 (26%)
LVEDD (mm)	65.2 (±7.91)
LVEF (%)	37.4 (±9.2)
LA index vol (mL/m^2^)	42.1 (±18.8)
RV dilation (n, %)	14 (28%)
Acei or ARB (n, %)	41 (82%)
Beta-blocker (n, %)	44 (81.6%)
Diuretics (n, %)	31 (63.6%)
Digoxin (n, %)	12 (24%)
Amiodarone (n, %)	22 (44%)
BNP (pg/mL)	254 (154–445)
Ca-125 (U/mL)	9 (5.5–17.2)
Uric acid (mg/dL)	6.2 (±2)
CPR (mg/dL)	4.5 (4.5–7.3)
TNFα	2.5 (2.2–2.9)
IL-1β	3.6 (3.3–4.3)
IL-6	4.1 (3.6–7.7)
IL-8	6.8 (5.7–11.3)
IL-10	2.5 (2.2–2.7)
IL-12	2.7 (2.5–2.9)
Adverse event (n, %)	18 (36%)
Death (n, %)	6 (12%)

NYHA: New York Heart Association functional class, LVEF: left ventricular ejection fraction, LVEDD: Left ventricular end-diastolic diameter, LA index vol: left atrial index volume, RV dilation: right ventricular dilation, DT: deceleration time, BNP: natriuretic peptide type B, CPR: C- reactive protein, TNFα: tumor necrosis factor α, IL6: interleukin 6, IL-8: interleukin 8, IL-10: interleukin 10, IL-1β: interleukin1β, Acei: angiotensin-converting enzyme, ARB: angiotensin receptor blocker. Results are reported as mean ± SEM or as median (25th and 75th percentiles) or for categorical variables (% of total patients in brackets).

**Table 2:** Echocardiography, laboratorial and clinical parameters and adverse cardiac events.

	Event-Free (32 Patients—64%)	Adverse Cardiac Event (18 Patients—36%)	*p*
LVEDD mm	63 (±8.1)	69 (±5.7)	0.007
LVESD mm	50.5 (±9.7)	57 (±6.8)	0.016
DT ms	218 (185–283)	187 (156–228)	0.064
PSAP mmHg	31.5 (26.2–38.7)	35 (24.5–39)	0.4
E/e’	8.4 (5.8–11.9)	10 (5.4–13)	0.7
LA index volume mL/m^2^	36.6 (±12.5)	54.5 (±24.7)	0.003
BNP pg/mL	201 (93–374)	392 (237–911)	0.004
Ca-125 U/mL	8.5 (5.5–14,5)	11 (5.8–24)	0.4
Uric acid mg/dL	5.9 (±1.8)	6.7 (±2.3)	0.2
C-reactive protein mg/dL	4.5 (4.5–6.9)	4.5 (4.5–8.8)	0.5
Albumin g/dL	4.3 (±0.3)	4.08 (±0.4)	0.038
Sodium meq/L	141 (139–142)	139 (138–141)	0.14
Use of amiodarone	31.2%	66.7%	0.017
Pulmonary rales	12.9%	38.9%	0.042
RV dilation	15.6%	50%	0.019
TNFα	2.57 (2.3–2.9)	2.5 (2.1–14.6)	0.8
IL-1β	3.58 (3.3–3.8)	3.7 (3.5–6.8)	0.3
IL-6	4.01 (3.5–5.7)	6 (3.7–11.3)	0.1
IL-8	6.5 (5.7–7.6)	9.9 (6.1–26.4)	0.047
IL-10	2.44 (2.1–2.7)	2.68 (2.25–4.23)	0.29
IL-12	2.73 (2.43–2.92)	2.9 (2.7–3.6)	0.019

LVEDD: left ventricle end-diastolic diameter, LVESD: left ventricle systolic diameter, DT deceleration time, PSAP: pulmonary artery systolic pressure, E/e’: ratio of early transmitral velocity to tissues Doppler mitral annular early diastolic velocity, LA index volume: left atrial index volume, RV dilation: right ventricular dilation. Results are reported as mean ± SEM or as median (25th and 75th percentiles) or for categorical variables (% of total patients in brackets).

**Table 3:** Echocardiography, laboratorial and clinical variables and survival.

Variables	Survival	Death	Sig
LVEDD mm	64 (±8.1)	69 (±4.6)	0.14
LVESD mm	52 (±9.4)	58 (±6.5)	0.14
DT ms	211 (183–281)	151 (120–183)	0.08
PSAP mmhg	32 (26–39)	36 (32–46)	0.15
E/e’	8.7 (5.7–12)	13 (5.6–21)	0.69
LA index vol mL/m^2^	41 (±18)	60 (±0.7)	0.16
BNP pg/mL	221 (119–392)	790 (452–1063)	0.01
Ca-125 U/mL	8 (5.5–14.5)	20 (10.3–74)	0.056
Uric acid mg/dL	6 (±1.8)	7.7 (±2.8)	0.05
C-reactive protein mg/L	4.5 (4.5–7)	5.7 (4.5–18)	0.17
Albumim g/dL	4.2 (±0.4)	4.3 (±0.3)	0.7
Sodium meq/L	141(138–142)	140 (138–141)	0.6
RV dilation	22.7%	66.7%	0.044
Pulmonary rales	18.6%	60%	0.07
TNFα	2.5 (2.2–2.8)	10.5 (2.5–31)	0.1
IL-1β	3.6 (3.3–3.8)	8.53 (3.61–16)	0.08
IL-10	2.2 (2.2–2.7)	5 (2.1–14)	0.4
IL-6	3.6 (3.6–5.7)	10 (3.4–18)	0.3
IL-8	6.7 (5.7–8.7)	22.6 (6.3–245)	0.2
IL-12	2.7 (2.5–2.9)	4.2 (2.7–11.2)	0.069

LVEDD: left ventricle end-diastolic diameter, LVESD: left ventricle systolic diameter, DT: deceleration time, E/e’: ratio of ratio early transmitral velocity to tissues Doppler mitral annular early diastolic velocity, RV dilation: right ventricular dilation on echocardiogram, TNFα: tumor necrosis factor α, IL6: interleukin 6, IL-8: interleukin 8, IL-10: interleukin 10, IL-1β: interleukin1β. Results are reported as mean ± SEM or as median (25th and 75th percentiles) or for categorical variables (% of total patients in brackets).

**Table 4:** Univariate Cox regression model.

Variables	Significance	Estimated Risk	CI
Clinical			
NYHA	0.074	8.039	0.817–79.063
Hepatomegy/edema	0.19	2.919	0.588–14.482
Radiological congestion	0.179	4.384	0.508–37.814
Pulmonary rales	0.017	3.125	1.224–7.978
Ecocardiographic			
LVEDD	0.115	1.107	0.976–1.256
LA index volume	0.145	1.037	0.987–1.090
RV dilation	0.037	6.081	1.112–33.259
Laboratorial			
BNP pg/mL	0.558	1	0.999–1.002
CA-125 U/mL	0.054	1.010	1.000–1.067
Uric acid mg/dL	0.061	1.388	0.985–1.955
CPR mg/dL	0.054	1.157	0.997–1.343
TNFα	0.010	1.089	1.021–1.161
Il-1β	0.006	1.254	1.068–1.472
Il-12	0.009	1.330	1.074–1.646

NYHA: New York Heart Association functional class, Radiological congestion: presence of venous congestion on chest radiograph, La index volume: left atrial index volume, RV dilation: right ventricular dilation, LVEDD: left ventricle end-diastolic diameter, CPR: C-reactive protein.

**Table 5:** Multivariate Cox regression model.

Variables	Significance	Estimated Risk	CI
IL-1β	0.078	1.200	0.980–1.471
Pulmonary rales	0.104	2.391	0.835–6.842
RV dilation	0.849	1.252	0.123–12.765
IL-1β	0.006	1.254	1.068–1.472

## Data Availability

Data analyzed in this investigation are contained in this article. In need for more information please contact the authors.
